# Estimating Causal Effects of Malaria Infection on Anaemia Among Children Under Five Years in Uganda: Evidence From a Cross‐Sectional Malaria Indicator Survey

**DOI:** 10.1002/hsr2.73001

**Published:** 2026-08-05

**Authors:** Edson Mwebesa, Delight Mawufemor Agbi, Lameck Ondieki Agasa, Susan Awor, Herbert Tafadzwa Kazonda, Abraham Isiaka Jimmy, Scholastique M. Merveille Essetcheou, Isaac Kipkosgei Tum, Gregory Kibet Kerich, Ann Mwangi

**Affiliations:** ^1^ Muni University, Faculty of Science Arua Uganda; ^2^ School of Science and Aerospace Studies Moi University Eldoret Kenya; ^3^ School of Health Sciences Kisii University Kisii Kenya; ^4^ World Bank Development Research Group Kampala Uganda; ^5^ Department of Environmental Health Masvingo Polytechnic Masvingo Zimbabwe; ^6^ Department of Public Health University of Makeni Makeni Sierra Leone; ^7^ Laboratoire de Biomathématiques et d'Estimations Forestières Université d'Abomey‐Calavi Cotonou Bénin

**Keywords:** anemia, children under five, malaria, propensity score matched analysis, Uganda

## Abstract

**Background and Aims:**

Malaria and anemia remain major public health challenges contributing substantially to morbidity and mortality among children under 5 years in sub‐Saharan Africa (SSA). Although conventional regression analyses have reported a significant association between malaria infection and anemia, these approaches often inadequately account for socioeconomic and demographic confounders, thereby limiting causal interpretation. This study aimed to estimate the causal effect of malaria infection on anemia among children under five in Uganda using a robust quasi‐experimental approach.

**Methods:**

We conducted a Propensity Score Matched (PSM) analysis using data from the 2018–2019 Uganda Malaria Indicator (Cross‐Sectional) Survey (UMIS). The study population comprised children under 5 years of age with complete data on malaria status (exposure) and anemia status (outcome). A logistic regression model was used to estimate the propensity scores, incorporating a comprehensive set of child‐level, maternal, and contextual covariates. The Average Treatment Effect on the Treated (ATT) was estimated in Stata v14.0 using survey weights to account for the UMIS complex sampling design.

**Result:**

The prevalence of malaria infection among children under five was 23.6% [95% CI: 19.8, 27.8], while the prevalence of anaemia (mild, moderate, or severe) was 51.2% [95% CI: 48.3, 54.1]. The effect of exposure to malaria infection on anaemia was positive and significant [Average Treatment Effect of the Treated (ATT) = 25.6%, 95% CI: 17.6%, 33.5%, *p* < 0.001]. These results indicate that malaria infection increases the likelihood of anaemia by 25.5 percentage points among infected children under five in Uganda, after adjusting for key confounders.

**Conclusion:**

Malaria infection is a major driver of anaemia among Ugandan children under five. Strengthening malaria prevention and prompt treatment could substantially reduce childhood anemia and its associated health burden.

**Abbreviations**
ATT: Average Treatment Effect of the Treated
AU: African Union
CC: Common Caliper
CI: Confidence Interval
ICF: International Classification of Functioning, Disability and Health
ITN: Insecticide Treated Net
ITNs: Insecticide Treated Nets
LR: Likelihood Ratio
MIS: Malaria Indicator Survey
MM: Malaria Messages
NMCD: Uganda National Malaria Control Division
PSMA: Propensity Score‐Matched Analysis
SE: Standard Error
SMS: Short Messages
UBOS: Uganda Bureau of Statistics
UMIS: Uganda Malaria Indicator Survey
WHO: World Health Organization

## 1 Introduction

Malaria and anaemia remain leading causes of morbidity and mortality among children under five in tropical regions, especially in sub‐Saharan Africa [1, 2]. Malaria, caused by *Plasmodium falciparum* accounts for about 282 million cases and over half a million deaths worldwide, with the majority occurring in Africa [3]. Children under the age of five are disproportionately affected due to their underdeveloped immunity, which makes them highly vulnerable to severe disease and death [4]. Anaemia, defined as a haemoglobin concentration below 110 g/L in children under 5 years, had a global prevalence of 24.3% across all ages in 2021, corresponding to 1.92 billion cases [5, 6]. Anaemia is both a consequence of and a risk factor for malaria and contributes to child morbidity, impairs cognitive, social, and physical development, increases susceptibility to infections, and can lead to death in severe cases [7, 8].

The relationship between malaria and anaemia is complex and multifaceted. Malaria infection directly causes haemolysis of red blood cells, suppresses erythropoiesis in the bone marrow, and increases splenic clearance of both parasitized and non‐parasitized cells. These processes result in acute and chronic anaemia in children exposed to repeated or severe infections [9]. Conversely, pre‐existing anaemia increases the risk of severe malaria and worsens clinical outcomes [9]. Beyond biological mechanisms, social and environmental factors also shape the burden of malaria and anaemia in sub‐Saharan Africa. They include poor nutritional status, low maternal education, household poverty, inadequate sanitation, and limited access to preventive interventions such as insecticide‐treated nets, indoor residual spraying, and timely diagnosis and treatment [10, 11].

Uganda bears one of the highest malaria burdens globally, with transmission being perennial and intense in most regions [12]. The 2018–2019 Uganda Malaria Indicator Survey (UMIS) reported malaria prevalence of approximately 30% among children under five, with marked variation across regions [13]. Anaemia prevalence in this age group also remains alarmingly high, affecting more than 50% of children in some rural districts [14]. Despite substantial investments in malaria control, such as universal distribution of insecticide‐treated nets (ITNs), improved access to rapid diagnostic tests (RDTs), and artemisinin‐based combination therapies (ACTs), the dual burden of malaria and anaemia persists. This suggests that malaria continues to exert a strong effect on anaemia outcomes, but quantifying the magnitude of this relationship in representative population data remains a critical research priority.

Studies in Uganda and other African regions have consistently shown that malaria infection increases the risk of anaemia [15, 16, 17]. Despite this evidence, most analyses rely on conventional regression methods that inadequately control for socioeconomic, demographic, and environmental confounders to isolate the effect/impact from mere correlations. This limitation underscores the need for more robust approaches, such as propensity score matching, to better estimate the causal effect of malaria on anaemia using nationally representative data from the 2018–2019 UMIS. The UMIS provides nationally representative data that can be used to assess the relationship between malaria and anaemia. Propensity Score Matching (PSM) offers an advanced quasi‐experimental method that mimics randomization by balancing observed covariates between exposed and unexposed groups, enabling more rigorous estimation of causal effects [18]. Applying PSM to malaria‐related outcomes has strengthened causal inference in African survey‐based studies [19, 20, 21]. Thus, applying PSM to UMIS data offers an opportunity to generate robust and policy‐relevant evidence on the burden of malaria‐attributable anaemia while minimizing confounding bias. This study focuses on children under 5 years of age, a population that represents a critical window for growth, cognitive development, and survival. Understanding the extent to which malaria contributes to anaemia in this age group has direct implications for integrated disease control strategies. If malaria accounts for a significant proportion of anaemia cases, strengthening prevention and treatment efforts could substantially reduce childhood anaemia prevalence. This paper estimates the effect of malaria infection on anaemia among children under 5 years in Uganda using data from the 2018–2019 Malaria Indicator Survey (MIS).

## 2 Methodology

### 2.1 Data Source and Study Population

The study utilized data from the Uganda Malaria Indicator Survey (MIS) 2018–2019. Data were collected in 15 sub‐regions of Uganda and nine refugee settlements in the Northern and Western regions of the country. A two‐stage stratified sampling design was used to select 6915 children under 5 years. This process involved selecting clusters (enumeration areas) in stage one, followed by the systematic selection of households in the study. This study utilized Kids Recode data (for children under five). The children's dataset included data on interviewed women and children born 5 years before the survey in selected households. The sample comprised children under 5 years of age with complete data on malaria status (exposure) and anaemia status (outcome), yielding a sample of 4375 children. An elaborate methodology on Uganda MIS can be found in the MIS report [22].

### 2.2 Data Analysis

Data analysis involved estimating the prevalence of malaria and anaemia and describing baseline characteristics using proportions and their 95% confidence intervals for the study sample. Cross‐tabulations were applied to identify the distribution of malaria infection by sample characteristics. A 5% level of significance was used to identify significant impact or associations. Stata Version 14.0 was utilized for the analysis. Estimates were survey‐weighted to account for the complex sampling design in MIS data.

### 2.3 Propensity Score‐Matched Analysis (PSMA)

To assess the impact of malaria infection on anaemia in children under 5 years in Uganda, we employed a propensity score‐matched analysis (PSMA). This statistical technique is used to estimate the causal effects of malaria infection (exposure) in the absence of a clinical trial, by accounting for confounding variables [23, 24]. A propensity score is the probability that a child i, where (i = 1, 2, …, n) is exposed to malaria infection (the treatment group) or not exposed (the control group), given a set of measured covariates “X” [25]. The propensity score represents each child's estimated likelihood of having malaria based on their observed characteristics. Children with similar propensity scores are expected to have similar observed characteristics, allowing meaningful comparisons between children with and those without malaria. The propensity score is given in Equation 1 as;

$$e\left(X\right)=\text{Pr}\left(Z_{i}=1|X_{i}\right)$$

which shows that e(X) is the propensity score, which is the conditional probability of exposure to malaria infection, given a set of observed covariates $X_{i}$, $Z_{i}$ is the intervention variable with value 0 for the unexposed children and value 1 for the exposed children. The vector $X_{i}$ is associated with exposure to malaria infection [24, 26]. The use of PSMA is appropriate when observational data is available, and a randomized trial is unethical, impractical, or expensive or impossible, and there is a need to estimate the impact of interventions, especially at the population level [27].

This approach calculates propensity scores for each child based on observed covariates, addressing confounding and minimizing bias [23]. In this study, the analysis focused on obtaining the Average Treatment Effect of the Treated (ATT), which is the effect for children under 5 years who were exposed to malaria infection related to anaemia (measured as whether the child under five had anaemia or not) [24]. To ensure appropriate comparisons between children, we used nearest neighbour with a caliper matching (with a tolerance of 0.03) strategy based on the age of the woman or caregiver, the education level of the woman or caregiver, the household wealth index, household water sources, type of place of residence and age of the child.

To estimate the propensity scores, since the treatment variable (exposure to malaria) was binary, we used a binary logistic regression. The obtained scores were then matched for exposed and unexposed children, employing a caliper of 0.03, to match exposed and unexposed children [20, 24, 25]. The ATT is framed as a counterfactual in Equation 2 below.

$$\text{ATT}\;=E[Y_{i}\left(1\right)-Y_{i}\left(0\right)\;|Z_{i}=1]$$

where $Y_{i}\left(1\right)$ is the outcome in the malaria infection exposed group, $Y_{i}\left(0\right)\;$is the outcome in the malaria infection unexposed group, $E[Y_{i}\left(1\right)|Z_{i}=1]$ is the expected outcome of anaemia, assuming that all children under five have been exposed to malaria infection, and $E[Y_{i}\left(0\right)|Z_{i}=1]$ is the expected outcome of anaemia, assuming that all children under five exposed to malaria infection have not been exposed to malaria infection (counterfactual or unobserved) [23, 24]. The ATT is the expected difference in anaemia prevalence between children who were exposed to malaria infection and the same children if they had not been exposed (counterfactual) [26].

**Figure 1:**
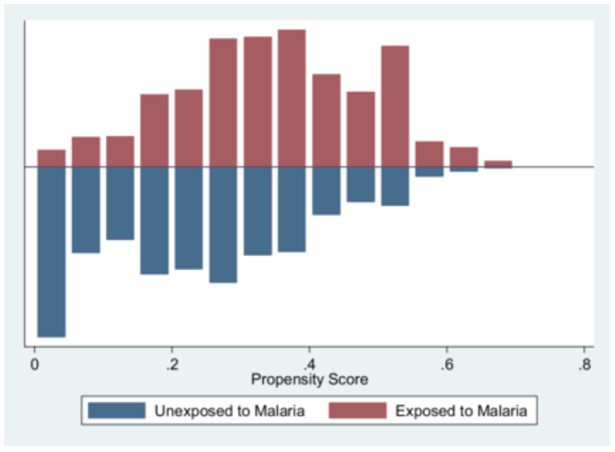
Distribution of estimated propensity scores for children under five in Uganda by malaria exposure status (1 = *Exposed*, 0 = *Unexposed*). The *x*‐axis shows the estimated propensity score (i.e., the probability of malaria exposure given observed covariates). The *y*‐axis shows the density of children at each score value. The overlap in propensity score distributions between exposed and unexposed groups supports the validity of the matching procedure.

The ATT were estimated using these steps: 1) selection of appropriate observed covariates for the propensity score estimation; 2) estimation of the propensity score; 3) checking for overlap and balance in the propensity scores; 4) computing the ATT [27]. The counterfactual scenario was established by matching children under 5 years who were exposed to malaria with unexposed children, based on observed covariates. We evaluated the overlap and balance of propensity scores between exposed children and unexposed children using propensity score graphs (Figure 1). Bootstrapping was used to estimate the standard errors and confidence intervals for the ATT. The off‐support children were excluded from the analysis to avoid biased parameter estimates and unstable treatment effects [28]. The psmatch2 command in Stata v14.0 was used to estimate treatment effects for malaria exposure and anaemia among children under 5 years in Uganda. All statistical tests were two‐sided at 5% level of significance. The reporting of statistics in this study followed guidelines suggested by [29].

### 2.4 Variables

**Treatment** $Z_{i}$ **:** The exposure to malaria infection in a child under 5 years. Exposure to malaria infection was defined as having malaria during the survey (*Yes* = 1, *No* = 0). Malaria positivity was a binary variable indicating detection of Plasmodium parasites by either a rapid diagnostic test (RDT) or microscopy. Malaria positivity was determined using a two‐step approach. A malaria rapid diagnostic test (mRDT; SD BIOLINE Malaria Ag Pf, Abbott) was performed, and positive cases were confirmed by Giemsa‐stained thick and thin blood smear microscopy, following WHO standard protocols [30].

**Outcome** $Y_{i}$ **:** This study outcome was anaemia, defined as having an altitude‐adjusted haemoglobin concentration of less than 11.0 g/dL (*Yes* = 1, *No* = 0). Haemoglobin concentration was measured from capillary blood obtained by finger or heel prick using a HemoCue Hb 201+ photometer, in line with the standard UMIS protocol [31].

**Baseline Characteristics** $X_{i}$ **:** These include woman's age (15–24 years, 25–29 years, 30 or more years), woman's education level (no education, primary, secondary, higher), household wealth index (poorest, poorer, middle, richer, richest), water source (improved, unimproved), type of place residence (urban, rural, refuge), age of the child (<1 year, 1–3 or more years) and mosquito net use by children under 5 years (no, some/all). These baseline characteristics were the ones used in the estimation of the propensity scores and upon which children were matched.

## 3 Results

The analysis of data from the 2018–2019 Uganda MIS for children under five is presented in this section. To account for confounding factors and calculate the causal relationship between malaria infection (exposure) and anaemia (outcome), a propensity score matching technique was utilized. Furthermore, contextual factors, mother characteristics, and child‐level factors are also presented in proportions and their 95% confidence intervals. Model diagnostics such as Pseudo R‐squared, Chi‐square, and bias metrics were used to evaluate the quality of the matching. The impact of malaria exposure on the prevalence of anaemia and the ATT was calculated and all presented.

### 3.1 Prevalence of Malaria and Associated Factors

The analysis revealed a malaria prevalence of 23.6% [95% CI: 19.8%, 27.8%] among children under 5 years in Uganda. This indicates that about 24 in every 100 children under 5 years were malaria positive during the survey. The prevalence of anaemia (mild, moderate or severe) was 51.2% [95% CI: 48.3, 54.1] among children under 5 years in Uganda. Several factors were analysed to determine their association with malaria infection. Among child‐level factors, the age of the child was significantly associated with malaria infection (*p* = 0.008). Children under 1 year had a lower malaria prevalence [20.1%, 95% CI: 16.1%, 24.8%] compared to children aged 1–2 years [23.2%, 95% CI: 19.1, 27.8] and those who were 3 years or older [25.9%, 95% CI: 21.6%, 30.7%]. This suggests that older children may be more exposed to malaria vectors, possibly due to increased outdoor activities or reduced use of protective measures. The use of mosquito nets was also significantly associated with malaria infection (*p* = 0.002), with children who slept under a mosquito net (some or all of them) showing a lower malaria prevalence [21.4%, 95% CI: 18.0%, 25.3%] compared to those who did not [30.9%, 95% CI: 23.7%, 39.1%]. No significant association was found between the child's sex (*p* = 0.892) or living arrangement (*p* = 0.661) and malaria infection.

Some maternal factors were also associated with malaria prevalence. The mother's education level was highly associated with malaria infection (*p* < 0.001). Children of mothers with no education had a higher malaria prevalence [39.4%, 95% CI: 31.5%, 48.0%] compared to those whose mothers had primary [25.3%, 95% CI: 20.9, 30.2], secondary [8.7%, 95% CI: 6.0%, 12.3%] or higher education [9.0%, 95% CI: 3.7%, 20.5%]. Maternal age showed no significant association with malaria infection (*p* = 0.559).

Household wealth index was strongly associated with malaria infection (*p* < 0.001), with children in the poorest households having the highest malaria prevalence [39.7%, 95% CI: 32.2%, 47.7%] compared to those in the richest households [2.8%, 95% CI: 1.5%, 5.3%]. The type of place of residence was highly significant (*p* < 0.001), with rural [26.3%, 95% CI: 21.5%, 31.8%] and refugee [42.5%, 95% CI: 28.9%, 57.3%] populations showing higher malaria prevalence compared to urban areas [7.0%, 95% CI: 3.7%, 12.9%]. Regional variations were also significant (*p* < 0.001), with the Northern region having the highest malaria prevalence [35.7%, 95% CI: 27.8%, 44.3%] and the Central region the lowest [2.4%, 95% CI: 1.1%, 5.4%]. The sex of the household head and source of drinking water showed no significant association with malaria infection (*p* = 0.085 and *p* = 0.420, respectively). The results are shown in Table 1.

**Table 1:** Prevalence of malaria and its related factors/covariates.

		Malaria		
		Negative	Positive	
	Overall	% [95% CI], row	% [95% CI], row	*p*‐value
	% [95% CI]	76.4 [72.2, 80.2]	23.6 [19.8, 27.8]	
**Prevalence of anaemia**				
Not anaemic	48.8 [45.9, 51.7]	84.9 [81.6, 87.8]	15.1 [12.2, 18.4]	
Anaemic	51.2 [48.3, 54.1]	67.9 [61.5, 73.7]	32.1 [26.3, 38.5]	< 0.001
**Child Factors**				
**Current age of the child**				
< 1 year	21.4 [19.8, 23.1]	79.9 [75.2, 83.9]	20.1 [16.1, 24.8]	0.008
1−2 years	39.1 [37.0, 41.1]	76.8 [72.2, 80.9]	23.2 [19.1, 27.8]	
3 or more	39.6 [38.0, 41.1]	74.1 [69.3, 78.4]	25.9 [21.6, 30.7]	
**The child slept under a mosquito net**				
No	25.1 [22.6, 27.9]	69.1 [60.9, 76.3]	30.9 [23.7, 39.1]	0.002
Some/all	74.9 [72.1, 77.4]	78.6 [74.7, 82.1]	21.4 [18.0, 25.3]	
**Mother level factors**				
**Age of the mother**				
15−24	26.5 [23.6, 29.5]	78.1 [72.6, 82.7]	21.9 [17.3, 27.4]	0.559
25−29	26.0 [23.2, 28.9]	74.5 [68.4, 79.8]	25.5 [20.2, 31.6]	
30+	47.6 [43.1, 52.1]	76.5 [71.1, 81.2]	23.5 [18.8, 29.0]	
**Highest education level**				
No education	19.6 [16.9, 22.5]	60.6 [52.0, 68.5]	39.4 [31.5, 48.0]	< 0.001
Primary	53.6 [50.1, 57.1]	74.8 [69.8, 79.1]	25.3 [20.9, 30.2]	
Secondary	22.5 [19.6, 25.8]	91.4 [87.7, 94.0]	8.7 [6.0, 12.3]	
Higher	4.3 [3.0, 6.1]	91.0 [79.5, 96.4]	9.0 [3.7, 20.5]	
**Contextual factors**				
**Wealth index**				
Poorest	29.3 [25.7, 33.2]	60.3 [52.4, 67.8]	39.7 [32.2, 47.7]	< 0.001
Poorer	21.0 [18.4, 23.9]	72.8 [65.8, 78.9]	27.2 [21.1, 34.2]	
Middle	16.7 [13.8, 20.0]	77.2 [70.2, 82.9]	22.8 [17.1, 29.8]	
Richer	15.2 [12.9, 17.8]	87.2 [81.6, 91.3]	12.8 [8.7, 18.4]	
Richest	17.8 [12.5, 24.8]	97.2 [94.7, 98.5]	2.8 [1.5, 5.3]	
**Source of drinking water**				
Improved	77.9 [70.8, 83.8]	75.5 [70.9, 79.6]	24.5 [20.5, 29.1]	0.420
Unimproved	22.1 [16.3, 29.2]	79.7 [69.4, 87.1]	20.4 [12.9, 30.6]	
**Type of place residence**				
Urban	23.3 [17.1, 30.9]	93.0 [87.1, 96.3]	7.0 [3.7, 12.9]	< 0.001
Rural	65.9 [58.5, 72.5]	73.7 [68.2, 78.5]	26.3 [21.5, 31.8]	
Refugee	10.8 [8.1, 14.4]	57.5 [42.7, 71.1]	42.5 [28.9, 57.3]	
**Region**				
Central	17.6 [12.9, 23.4]	97.6 [94.6, 98.9]	2.4 [1.1, 5.4]	< 0.001
Eastern	33.1 [27.9, 38.8]	74.2 [64.2, 82.2]	25.8 [17.8, 35.8]	
Northern	16.7 [14.2, 19.6]	64.4 [55.7, 72.2]	35.7 [27.8, 44.3]	
Western	32.6 [28.5, 37.1]	73.5 [67.5, 78.7]	26.5 [21.3, 32.5]	

### 3.2 Effect of Malaria Exposure on Anaemia Among Children Under 5 Years in Uganda

The figure (Figure 1) illustrates a propensity score graph showing the distribution of propensity scores for children under five in Uganda, categorized as either unexposed (blue) or exposed (red) to malaria infection. The overlap in propensity score distributions between the exposed and unexposed groups supports the validity of the matching process. The quality of matching, as shown in Table 3, improved significantly after PSM. The unmatched sample had a Pseudo *R* ^2^ of 0.115, a significant Likelihood Ratio (LR) Chi^2^ (561.83, *p* < 0.001), and relatively high mean and median bias,16.9% and 14.1%, respectively, indicating imbalance between groups. After matching with a common caliper of 0.03, the Pseudo *R* ^2^ decreased to 0.002, the LR Chi^2^ became non‐significant (6.60, *p* = 0.968), and the mean and median bias reduced to 2.1 and 1.7, respectively, confirming high‐quality matching and balance between groups.

The ATT was estimated to quantify the causal effect of malaria infection on anaemia among children under five who were exposed to malaria. The ATT represents the difference in anaemia prevalence between the exposed group (malaria‐positive) and a matched unexposed group (malaria‐negative) with similar characteristics. As shown in Table 2, the estimated ATT was 25.5% [95% CI: 17.0%, 33.9%, *p* < 0.001], indicating that malaria infection increased the likelihood of anaemia by 25.5 percentage points among exposed children after controlling for observed confounding through propensity score matching. Table 3 presents the matching diagnostics, which demonstrated substantial improvement in covariate balance following matching (Pseudo *R* ^2^ = 0.002; mean bias = 2.1%; median bias = 1.7%), supporting the robustness of the estimated treatment effect. Balance among all the variables (covariates) considered in this study was assessed and fell within required bounds. The results are shown in File S1.

**Table 2:** Estimation of ATT of exposure to malaria on anaemia among children under five in Uganda.

Intervention	Sample	Exposed	Unexposed	Difference	SE	*t*‐stat
Child exposed to Malaria	Unmatched	0.692	0.453	0.240	0.017	14.08
	ATT	0.692	0.438	0.255	0.044	5.74

Abbreviations: ATT, Average Treatment Effects of the Treated; SE, Standard Errors.

**Table 3:** Quality of matching and ATT of exposure to malaria on anaemia among children under five in Uganda.

		Model Diagnostics					ATT [95% CI]
Intervention	Sample	Pseudo *R* ^2^	LR Chi^2^	p > Chi^2^	Mean Bias	Median Bias	
Child exposed to Malaria	Unmatched	0.115	561.83	0.000	16.9	14.1	
	CC (0.03)	0.002	6.60	0.968	2.1	1.7	25.5% [17.0%, 33.9%]^a^

Abbreviations: ATT, Average Treatment Effect of the Treated, CC, Common caliper; Chi^2^, Pearson's Chi‐square; CI, Confidence Interval; LR, Likelihood Ratio.

^a^ *p* < 0.001.

## 4 Discussion

This study examined the causal effect of malaria infection on anaemia among children under 5 years in Uganda using propensity score matching applied to data from the 2018–2019 Uganda Malaria Indicator Survey. By estimating the average treatment effect on the treated, the analysis approximated the counterfactual risk of anaemia among malaria‐infected children had they not been infected. The findings indicate that malaria infection increases the probability of anaemia by 25.6%, suggesting a substantial and clinically meaningful effect. This magnitude is epidemiologically significant in a setting where malaria and anaemia remain highly prevalent public health concerns. The observed effect is biologically plausible and consistent with existing evidence from malaria‐endemic regions. Notably, the estimated effect size is comparable to, and in some cases exceeds, associations reported in conventional regression‐based studies conducted in high‐transmission settings, thereby reinforcing the public health significance of malaria as a major driver of paediatric anaemia [32, 33].

Malaria contributes to anaemia through haemolysis of infected and uninfected red blood cells, splenic sequestration, dyserythropoiesis, and bone marrow suppression [9, 34, 35]. These mechanisms disproportionately affect young children whose immune systems and iron stores are still developing [36]. Studies conducted in sub‐Saharan Africa have consistently demonstrated that malaria is a major contributor to paediatric anaemia, particularly in high‐transmission settings [15, 36, 37, 38]. By explicitly balancing observed confounders between infected and non‐infected children, the propensity score matching approach enhances internal validity and reduces model dependence relative to traditional multivariable regression, thereby strengthening support for a causal interpretation of the relationship. Under the assumptions of conditional exchangeability and adequate covariate overlap, the matched estimates provide more robust evidence of effect than conventional associative models.

The findings also highlight important socioeconomic and geographic disparities. Children residing in poorer households, rural areas, refugee settlements, and the Northern region exhibited higher malaria prevalence. These patterns reflect structural inequalities in housing quality, environmental exposure, access to preventive tools such as insecticide‐treated nets, and availability of health services. Regional heterogeneity in malaria burden in Uganda has previously been attributed to climatic conditions, vector ecology, and infrastructural limitations [39, 40]. The elevated prevalence observed in the Northern region underscores the need for geographically targeted control strategies that combine preventive measures, early diagnosis, and effective treatment. Importantly, these structural disparities likely amplify the estimated causal effect by increasing both exposure intensity and the probability of recurrent infections, thereby compounding the risk of malaria‐related anaemia among socioeconomically disadvantaged children.

The protective association observed among children who slept under mosquito nets reinforces the importance of sustained malaria prevention efforts. Insecticide‐treated nets have been shown to significantly reduce malaria incidence in endemic areas, and their expanded coverage may indirectly reduce anaemia by lowering infection rates [41]. Strengthening distribution systems, promoting consistent use, and prioritizing vulnerable populations such as rural households and displaced communities remain critical components of malaria control policy. Integrated management approaches that incorporate routine hemoglobin screening for children diagnosed with malaria could further mitigate adverse outcomes. Early identification and treatment of anaemia alongside antimalarial therapy may prevent complications and support improved growth and cognitive development. Such integrated strategies may be particularly cost‐effective in high‐burden regions where the dual prevalence of malaria and anaemia creates overlapping health risks.

Malaria‐related anaemia has implications that extend beyond acute illness. Childhood anaemia has been associated with impaired cognitive development, reduced school performance, increased susceptibility to infections, and elevated mortality risk [42, 43]. Therefore, reducing malaria transmission may contribute not only to immediate reductions in morbidity but also to long‐term improvements in human capital and national development outcomes. Interventions targeting malaria in early childhood may yield sustained developmental benefits through their impact on haemoglobin levels and overall child health. From a broader development perspective, mitigating malaria‐related anaemia may enhance educational attainment and productivity trajectories, thereby generating intergenerational socioeconomic gains.

Despite the strengths of this study, several limitations warrant consideration. Propensity score matching adjusts only for observed covariates, and residual confounding from unmeasured factors such as nutritional deficiencies, genetic haemoglobinopathies, or helminth infections may persist. The cross‐sectional design limits the ability to establish temporality with certainty, although biological evidence strongly supports malaria as a cause of anaemia. Measurement error in rapid diagnostic tests for malaria or haemoglobin assessment could introduce misclassification. In addition, the validity of the matching approach depends on the positivity assumption, and limited overlap in certain subgroups may influence the stability of the estimated treatment effect. Sensitivity analyses exploring alternative matching specifications or doubly robust estimators could further strengthen causal inference. Future research using longitudinal data or quasi‐experimental designs may provide deeper insight into the robustness and temporal dynamics of the observed effect.

## 5 Conclusion

This study assessed the causal effect of malaria infection on anaemia among children under 5 years in Uganda using propensity score matching applied to the 2018–2019 Uganda Malaria Indicator Survey. The findings provide robust evidence that malaria infection substantially increases the likelihood of anaemia among exposed children. The estimated average treatment effect on the treated indicates that malaria infection raises the probability of anaemia by 25.6 percentage points, demonstrating that malaria is a major driver of the persistent burden of childhood anaemia in Uganda.

These results carry important public health implications. Integrating malaria control with anaemia prevention strategies within child health programs is essential. Expanding universal access to insecticide‐treated nets, strengthening indoor residual spraying, and ensuring prompt diagnosis and effective antimalarial treatment are likely to yield dual benefits by reducing both malaria transmission and anaemia prevalence. Routine haemoglobin screening for malaria‐positive children may further support early detection and management of anaemia, particularly in high‐transmission and socioeconomically disadvantaged regions such as Northern Uganda.

Although propensity score matching enhances causal inference by balancing observed confounders, the cross‐sectional design limits definitive conclusions about temporality, and unmeasured factors such as nutritional deficiencies or genetic haemoglobinopathies may still influence the association. Longitudinal studies and advanced causal methods could provide additional insight into the dynamic relationship between malaria and anaemia. Nevertheless, the evidence presented underscores that effective malaria control represents a critical strategy for reducing childhood anaemia and improving child health outcomes in Uganda.

## Author Contributions

**Edson Mwebesa:** methodology, conceptualization, data curation, writing – original draft, formal analysis, writing – review and editing. **Delight Mawufemor Agbi:** writing – review and editing, conceptualization, formal analysis, data curation, methodology. **Lameck Ondieki Agasa:** writing – review and editing, methodology, data curation. **Susan Awor:** writing – review and editing, software, data curation. **Herbert Tafadzwa Kazonda:** writing – review and editing, software. **Abraham Isiaka Jimmy:** writing – review and editing, data curation, methodology. **Scholastique M. Merveille Essetcheou:** writing – review and editing, data curation. **Isaac Kipkosgei Tum:** supervision, validation, writing – review and editing. **Gregory Kibet Kerich:** supervision, validation, writing – review and editing. **Ann Mwangi:** supervision, validation, writing – review and editing.

## Funding

The authors have nothing to report.

## Ethics Statement

The data used in this study are publicly available secondary data obtained from the DHS Program upon an approved data access request. Ethical approval was not required for this secondary analysis of anonymised, publicly available data. The original UMIS data collection was approved by the Uganda National Council for Science and Technology and the ICF Institutional Review Board.

## Conflicts of Interest

The authors declare no conflicts of interest.

## Transparency Statement

Edson Mwebesa affirms that this manuscript is an honest, accurate, and transparent account of the study being reported; that no important aspects of the study have been omitted; and that any discrepancies from the study as planned have been explained.

## Supporting information


Supporting File

## Data Availability

The datasets analysed during the current study were obtained from MEASURE DHS program.
