# Supplementary material for: Estimating Causal Effects of Malaria Infection on Anaemia Among Children Under Five Years in Uganda: Evidence From a Cross‐Sectional Malaria Indicator Survey
Source: Health Sci Rep. 2026 Aug 5;9(8):e73001. doi: 10.1002/hsr2.73001 (PMC13443140; doi:10.1002/hsr2.73001)
Supplement: Supplementary file 1 [file HSR2-9-e73001-s001.docx]

**Supplementary File 1**

**Table 1: covariate balance**

Figure 1. Love Plot Showing Standardized Mean Differences Before and After Matching

Table 2: Sensitivity Analysis of the ATT using Rosenbaum Bounds

| **Gamma** | **Q_mh+** | **Q_mh-** | **p_mh+** | **p_mh-** |
| --- | --- | --- | --- | --- |
| 1.0 | 37.1262 | 37.1262 | <0.001 | <0.001 |
| 1.5 | 32.3762 | 43.0216 | <0.001 | <0.001 |
| 2.0 | 29.529 | 48.0475 | <0.001 | <0.001 |
| 2.5 | 27.5678 | 52.5294 | <0.001 | <0.001 |
| 3.0 | 26.1025 | 56.6225 | <0.001 | <0.001 |
